# Production of *Aloe vera* Phytoplacenta Extract and Potential Applications in Skincare

**DOI:** 10.3390/life15030397

**Published:** 2025-03-03

**Authors:** Seung Min Jung, Hye-In Kim, Soo-Yun Kim, Sung Joo Jang, Hyo Hyun Seo, Jeong Hun Lee, Ju-Duck Kim, Won Kyong Cho, Sang Hyun Moh

**Affiliations:** 1 Department of Beauty Industry, Sungshin Women’s University, Seoul 02844, Republic of Korea; im0826se@gmail.com (S.M.J.); jdkim303@sungshin.ac.kr (J.-D.K.); 2 Plant Cell Research Institute of BIO-FD&C Co., Ltd., Incheon 21990, Republic of Korea; hikim@biofdnc.com (H.-I.K.); sykim@biofdnc.com (S.-Y.K.); sjjang@biofdnc.com (S.J.J.); hhseo@biofdnc.com (H.H.S.); jhlee@biofdnc.com (J.H.L.)

**Keywords:** *Aloe vera* phytoplacenta extract, skincare, moisturizing, wound healing, anti-inflammatory, RNA sequencing

## Abstract

*Aloe vera* has garnered significant scientific and commercial attention due to its multifaceted therapeutic and cosmetic potential. This study aimed to investigate the biological effects and molecular mechanisms of *Aloe vera* phytoplacenta extract (AVPE) on HaCaT cells and skin health. To achieve this, we investigated AVPE, produced using advanced in vitro cell culture techniques, and its effects on HaCaT cells. At 2% concentration, AVPE demonstrated remarkable biological effects, increasing AQP3 protein expression by 120% and healing area fourfold while simultaneously reducing *COX-2* messenger RNA (mRNA) by 43% and *iNOS* mRNA by 48%. An AVPE-containing product notably reduced facial skin temperature to 24.9 °C compared to 32.3 °C for the control product. RNA-sequencing (RNA-seq) analysis of transcriptional changes in HaCaT cells after AVPE treatment revealed 14 upregulated and 58 downregulated RNAs. Upregulated processes included response to hydrogen peroxide and muscle cell migration, while downregulated processes involved cell–cell adhesion and synaptic transmission. Pathway analysis further highlighted significant metabolic changes, including upregulation of pentose phosphate and galactose metabolism pathways and downregulation of the leishmaniasis and GABAergic synapse pathways. In addition, gene expression data indicated subtle changes in epidermal differentiation genes, modulation of inflammatory markers, and alterations in genes related to cell signaling and skin-specific functions. Our comprehensive findings underscore AVPE’s potential in enhancing skin healing, regulating temperature, and modulating cellular processes.

## 1. Introduction

*Aloe vera* L., scientifically known as *Aloe barbadensis* Miller, is a succulent plant originating from Western Asia, particularly the Arabian Peninsula [1,2]. It belongs to the Asphodelaceae family [3] and is widely cultivated in subtropical regions, including areas of the United States, Mexico, and India [1,2,4]. The plant is known by numerous common names, such as medicinal aloe, burn plant, and true aloe. The plant itself is a clump-forming succulent with fleshy gray–green leaves arranged in a vase-shaped rosette, typically growing up to 2 feet high and producing small yellow or orange flowers in winter and spring. Its versatility and natural healing properties have made it a popular plant in traditional medicine across various cultures, including those in Egypt, Greece, Mexico, India, China, and Japan [5].

*Aloe vera* has garnered significant scientific and commercial attention due to its multifaceted therapeutic applications and cosmetic potential. The viscous, gel-like substance extracted from its leaves is widely applied to address various dermatological issues, including sunburn, minor burns, acne, and skin irritations [1,2,5,6]. The plant’s remarkable healing capabilities are attributed to its rich composition, which includes over 20 minerals, essential amino acids, and a range of vitamins such as A, B1, B2, B6, B12, C, and E2 [7]. Furthermore, *Aloe vera* contains a diverse array of phytochemical components, including tannins, alkaloids, flavonoids, and phenols [8]. These compounds contribute to the plant’s potent antioxidant and anti-inflammatory properties, which have been demonstrated in both laboratory and living organism studies [9]. Beyond its well-known applications in skincare, *Aloe vera* has shown promise in various other areas of health and wellness. For example, research has indicated its possible antibacterial [10,11], anti-diabetic [12], antioxidant [13], anti-inflammatory [14], wound-healing [15], hepatoprotective [16], and even anticancer applications [17], highlighting the plant’s versatility and potential in modern medicine and healthcare.

In plants, the placenta is a nutrient-dense tissue within the ovary to which the ovules and developing seeds attach [18]. This mechanism supports seed development and reflects fundamental reproductive strategies across life forms. Phytoplacenta, a plant-derived alternative to animal placenta, has gained traction in the cosmetic and healthcare industries [19]. Extracted from sources like soybeans and rye, it is rich in amino acids, vitamins, and minerals, and its applications span skincare (anti-aging and moisturizing), haircare (hair growth and scalp health), and systemic health (cardiovascular benefits) [19]. Phytoplacenta’s antioxidant properties and cell regeneration abilities make it valuable in various products. Phytoplacenta could be used in various skincare products, such as anti-aging serums, moisturizers, and face masks. In haircare, it has potential for incorporation into hair growth serums and scalp treatments. These applications would leverage phytoplacenta’s reported benefits in cell regeneration, moisturizing, and antioxidant properties.

Plant cell culture technology is a powerful approach for producing high-value compounds in cosmetics, pharmaceuticals, and food industries by cultivating plant cells in controlled environments [20,21,22]. Recent studies have demonstrated its potential for creating bioactive compounds. Ginsenosides and stem cell cultures have been successfully produced from plants such as *Panax vietnamensis* and *Calendula officinalis* [23,24]. Similarly, plant cell culture applications have shown promising results for various species, including *Hibiscus sabdariffa*, *Gynostemma pentaphyllum*, and *Leontopodium alpinum* [25,26,27]. This technology provides a cost-effective and simple biological process for the large-scale synthesis of plant-derived secondary metabolites. It also offers better yields and excellent scaling-up attributes compared to conventional whole-plant extraction methods.

In this study, we investigated the potential of *Aloe vera* phytoplacenta extract (AVPE) as an innovative skincare ingredient. Our research encompassed several key aspects: developing a novel in vitro method for AVPE production, evaluating AVPE’s effects on various aspects of skin health (including moisturizing, wound healing, and anti-inflammatory responses), performing a clinical trial to assess AVPE’s skin-soothing properties, and carrying out RNA-sequencing (RNA-seq) analysis on AVPE-treated human keratinocytes to determine its molecular-level effects. That is, we studied AVPE in different ways to understand how it could be used to create better skincare products in the future.

## 2. Materials and Methods

### 2.1. AVPE Production

The *Aloe vera* samples used in this study were obtained from plants cultivated on a farm in Korea. We modified the phytoplacenta extraction protocol originally developed for *Camellia japonica* to accommodate the unique properties of *Aloe vera* [19]. To extract the phytoplacenta tissue, the flower bottoms were cross-sectioned using a sterile scalpel in a laminar flow hood. The specific region targeted for extraction was the ovule and surrounding nucellar tissue. The extracted tissue was then cut into smaller fragments (0.5–1 cm) to enhance sterilization and subsequent culture initiation. A two-step sterilization process was employed. First, the tissue fragments were immersed in 70% ethanol for 30 s to eliminate surface contaminants, followed by a sterile distilled water rinse to remove residual ethanol. This step was followed by agitation in a 0.3% sodium hypochlorite solution for 20 min to further reduce microbial load. The tissue was then rinsed three times with sterile distilled water to remove all traces of sodium hypochlorite. This two-step sterilization process was aimed at ensuring effective microbial decontamination while minimizing damage to the delicate plant tissues.

Callus induction was performed on Murashige and Skoog (MS) medium supplemented with 1 mg/mL 6-benzylaminopurine (BAP) and 0.5 mg/mL 2,4-dichlorophenoxyacetic acid (2,4-D). The pH of the MS medium was adjusted to 5.8 using 1N NaOH before autoclaving at 121 °C for 15 min. Cultures were maintained in dark conditions at 25 ± 2 °C. Callus formation was visually monitored, and after 4 weeks, a vigorously growing callus line (selection criteria: friable, fast-growing, healthy appearance, light green color) was selected for further propagation. The selected callus line was transferred to a 3 L stirred bioreactor (BIO-FD&C Co., Ltd., Incheon, Korea) for suspension cell culture. After 2 weeks, the cultured suspension cells were transferred to a 20 L bioreactor operated with a working volume of 15 L of liquid MS medium supplemented with 1 mg/mL BAP and 0.5 mg/mL 2,4-D. The culture was maintained under the following conditions for 6 weeks: agitation speed 100 rpm, temperature 25 ± 2 °C, continuous darkness, and aeration rate 1 vvm (volume of air per volume of culture per minute). Cell growth was monitored by periodic visual inspection and cell counting using a hemocytometer.

After cultivation, the callus was harvested and thoroughly washed five times with sterile distilled water to remove any residual media components. The harvested callus was then dehydrated using a freeze-drying process at −50 °C and 0.1 mbar for 72 h. Next, the dried *Aloe vera* callus underwent a two-step extraction process. First, 10 g of the dried callus was stirred in 1 L of distilled water at 50 °C for 8 h at 100 rpm using a magnetic stirrer. This initial extraction was followed by a heat extraction in which the extract from the first step was combined with another 1 L of distilled water and heated to 98 °C for 1 h in a shaking water bath at 100 rpm. The resulting AVPE was then filtered through a 0.22 μm filter to remove any particulate matter, and the filtrate was stored at −20 °C until use.

### 2.2. Human Immortalized Keratinocytes Cell Culture

Human immortalized keratinocytes (HaCaT cell line), obtained from ATCC (Manassas, VA, USA), were maintained in Dulbecco’s modified Eagle medium (Welgene, Gyeongsan, Republic of Korea) enriched with 10% fetal bovine serum (FBS) (Thermo Fisher Scientific, Waltham, MA, USA) and a 1× antibiotic–antimycotic solution (Thermo Fisher Scientific). Cells were incubated at 37 °C in an atmosphere containing 5% CO_2_ for 24–48 h, depending on the specific experimental requirements, to allow for proper cell attachment and growth. Throughout the experimental period, HaCaT cells underwent 12 passages.

### 2.3. Assessment of AVPE’s Effect on Human Keratinocyte Viability Using Cell Counting Kit-8 (CCK-8) Assay

To assess the effects of AVPE on the growth and viability of human keratinocytes, we employed a colorimetric method using CCK-8 (Catalog Number CCK-3000, Donginbio, Seoul, Republic of Korea). This assay is based on the reduction of a water-soluble tetrazolium salt, WST-8 [2-(2-methoxy-4-nitrophenyl)-3-(4-nitrophenyl)-5-(2,4-disulfophenyl)-2H-tetrazolium, monosodium salt], to a yellow-colored formazan dye by dehydrogenases in viable cells, with the amount of formazan dye generated by cellular dehydrogenase activity being directly proportional to the number of living cells [28]. HaCaT cells were seeded into 96-well plates at a density of 50,000 cells per well and allowed to attach for a full day. The following day, varying concentrations of AVPE (0.5%, 1%, and 2%) were applied to the cells, with pure water being used as the control condition. After a 24 h exposure period, we added 10 μL of the CCK-8 reagent to each well containing 100 μL of medium and continued incubation for an additional 3 h at 37 °C. Using a Multiskan SkyHigh Microplate Spectrophotometer (Thermo Fisher Scientific), we measured the optical density at 450 nm, which corresponds to the absorbance peak of the formazan dye. To quantify cell viability, we expressed the results as a percentage, comparing the absorbance values of AVPE-treated cells to those of the control group. The experiment was conducted in triplicate to ensure reproducibility.

### 2.4. Evaluation of AVPE’s Moisturizing and Anti-Inflammatory Effects Using Real-Time Reverse Transcription Polymerase Chain Reaction (RT-PCR)

Real-time RT-PCR was used to examine the moisturizing and anti-inflammatory effects of AVPE. We performed real-time RT-PCR with known primers amplifying marker genes using QuantiTect Primer Assays according to the manufacturer’s instructions (Qiagen, Hilden, Germany) [29]. HaCaT cells were incubated in a 96-well plate for 24 h at a density of 5 × 10^4^ cells per well. The cells were then treated with final concentrations of 0.5%, 1%, and 2% AVPE for 24 h. cDNA was synthesized from the treated HaCaT cells using a SuperPrep Cell Lysis & RT Kit for qPCR according to the manufacturer’s instructions (Toyobo, Osaka, Japan). To evaluate the moisturizing effect of AVPE treatment, we examined the expression of the gene encoding Aquaporin 3 (AQP3) by real-time RT-PCR. For the anti-inflammatory assay, HaCaT cells were incubated and then irradiated with UVB using a CL-1000 UV box at 5 mJ/cm^2^ (Analytik Jena AG, Jena, Germany). The cells were subsequently treated with 0.5%, 1%, and 2% AVPE for 4 h. Dexamethasone at a final concentration of 1 μM was used as a positive control (Sigma-Aldrich, St. Louis, MO, USA). To assess the anti-inflammatory effect of AVPE treatment, we quantified the expression of genes encoding *COX-2* and *iNOS* by real-time RT-PCR. The expression of individual genes was normalized to *GAPDH* gene expression.

### 2.5. Quantitative Analysis of Epidermal Growth Factor (EGF)-Induced Wound Healing

To assess AVPE’s wound-healing capacity, we employed a specialized technique using culture inserts in 24-well plates. HaCaT cells were seeded into these inserts at a concentration of 3 × 10^5^ cells per well and allowed to grow for 24 h until they reached near-confluence (approximately 90%). Upon removal of the inserts, we captured initial images to document the starting cell configuration. The experimental groups and positive controls were then treated with 100 ng/mL of EGF. Following an 18 h incubation period, the cells were fixed using a 4% paraformaldehyde solution for 15 min. After rinsing with phosphate buffered saline (PBS), we took final images of the cell populations. To quantify the extent of wound healing, we utilized ImageJ software (version 1.54j) (https://imagej.net/ij/) (Accessed on 10 August 2024) to measure the cell migration distance between the initial (0 h) and final (18 h) time points.

### 2.6. Human Clinical Trial to Evaluate Soothing Effect of AVPE on Heat-Induced Skin Irritation

A clinical study was conducted to assess the soothing effect of AVPE on heat-induced skin irritation. The trial, approved by the Institutional Review Board of the Korea Testing & Research Institute (KTR-HR-22-0018), involved 22 healthy female participants aged 20–59 years. The test subject selection process for this clinical trial involved 22 healthy adult women aged 20–59, all of whom met specific inclusion criteria and did not fall under exclusion criteria. Inclusion criteria encompassed being free of skin diseases and chronic illnesses, voluntarily consenting to this study, and being available for follow-up. Exclusion criteria included pregnant or nursing women, those using steroid-containing topical products, individuals with allergies or sensitivities, and recent participants in similar studies. This study strictly adhered to Good Clinical Practice (GCP) guidelines and the Helsinki Declaration, ensuring subject safety and privacy. Subjects were thoroughly informed about potential adverse effects, and compensation measures were established. The study protocol included initial screening, obtaining informed consent, skin measurements before and after product application, and questionnaire evaluations. Importantly, no adverse reactions related to the use of the test product were reported during the entire study period.

The test product was a gel formulation, and this study utilized a FLUKE Ti300 Plus Thermal Camera to measure skin temperature changes. After a 30 min acclimatization period, the baseline skin temperature was recorded. An infrared radiator was then used to increase the skin temperature above normal levels. Next, the test product was applied to the right side of the face, while a control product was applied to the left side. Skin temperature was measured immediately after product application. Statistical analysis was performed using IBM SPSS Statistics (Version 25) (Armonk, NY, USA), employing parametric (paired *t*-test) or non-parametric (Wilcoxon signed rank test) methods based on normality tests. Significance was determined at *p* < 0.05 with a 95% confidence interval.

### 2.7. Total RNA Isolation and Library Preparation for High-Throughput Sequencing

Total RNA was extracted from cells using TRIzol reagent (Invitrogen, Waltham, MA, USA) according to the manufacturer’s guidelines. The quality of the extracted RNA was evaluated using Bioanalyzer 2100 (Agilent, Santa Clara, CA, USA), after which RNA samples with an RNA Integrity Number (RIN) of 7 or higher were selected for library preparation. Libraries were constructed using the TruSeq Stranded mRNA LT Sample Prep Kit as per the manufacturer’s protocol (Illumina, San Diego, CA, USA). Six libraries with their respective indices were sequenced using the HiSeq X system (Illumina) in paired-end mode.

### 2.8. Mapping and Analysis of RNA-Seq Reads for Gene Expression Profiling

The sequencing data generated in this study were deposited in the NCBI SRA database (accession numbers: SRR32174142–SRR32174147) [30]. Raw reads were aligned to the GRCh38 reference genome using BBMap with default parameters. DESeq2 in DEBrowser v1.24.1 was employed to identify differentially expressed genes (DEGs) based on the number of reads mapped to each transcript [31]. DESeq2 does implement a built-in correction for multiple testing using the Benjamini–Hochberg method to control the false discovery rate (FDR). The *p*-values were adjusted for multiple testing using the Benjamini–Hochberg method to control the false discovery rate. We compared AVPE-treated HaCaT cells with those treated with distilled water (control). Read counts were normalized using the MRE method without additional corrections. DEGs were identified using criteria of fold change (FC) greater than 2 and *p*-values less than 0.05. For the AVPE-All comparison, all six datasets from AVPE-treated samples were compared against the three control datasets.

### 2.9. Comprehensive Pathway Enrichment Analysis of Differentially Expressed RNAs (DERs) Using WebGestalt

KEGG and Reactome pathway enrichment analysis was performed using WebGestalt 2024 (WEB-based GEne SeT AnaLysis Toolkit) [32,33] using the DERs identified from RNA-seq data as input. We selected *Homo sapiens* as the organism of interest and chose both KEGG and Reactome as functional databases. Over-Representation Analysis (ORA) was conducted with the following parameters: minimum number of genes per category set to 5, maximum to 2000, and significance level at FDR < 0.05. The reference gene set was the human genome. WebGestalt’s redundancy reduction feature was applied to improve result interpretation. The tool provided enrichment scores, adjusted *p*-values, and pathway diagrams for significantly enriched pathways. Pathways with adjusted *p*-values < 0.05 were considered statistically significant. This analysis allowed us to identify key biological processes and pathways affected by AVPE treatment in human keratinocytes.

The identified enriched GO terms, KEGG pathways, and Reactome pathways for upregulated and downregulated RNAs were visualized using GO and pathway enrichment bubble plots generated by SRplot (https://www.bioinformatics.com.cn/) (Accessed on 15 January 2025) [34]. These bubble plots provided an intuitive representation of the enrichment analysis results, displaying the significance and gene count for each enriched term or pathway.

### 2.10. Analysis of Expression of Genes Related to Skin Health and Function

We examined the expression of genes associated with various aspects of skin health and function, including epidermal differentiation, inflammation, oxidative stress response, and cell signaling. The expression data for these genes were extracted from the normalized RNA-seq dataset. To visualize the expression patterns, we utilized bar graphs generated by SRplot.

## 3. Results

### 3.1. In Vitro Production and Extraction of Aloe vera Phytoplacenta

**Figure 1:**
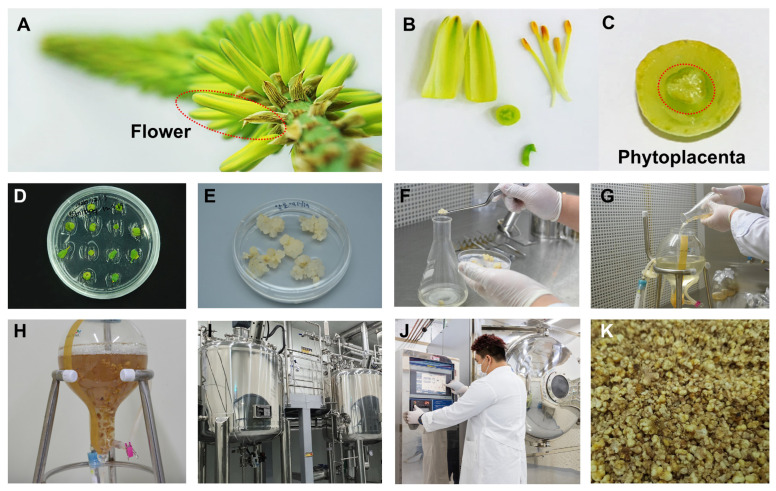
Production of *Aloe vera* phytoplacenta extract (AVPE) from flower to final product: (**A**) Close-up image of the *Aloe vera* flower, highlighting the structure and arrangement of the petals. (**B**) Dissected parts of the *Aloe vera* flower, including petals and stamens. (**C**) Cross-sectional view of the phytoplacenta, with the central part highlighted by a red dotted circle. (**D**) Multiple explants of *Aloe vera* phytoplacenta cultured in vitro. (**E**) The induced callus on MS medium. (**F**) Preparation of suspension cell culture of *Aloe vera* phytoplacenta cells in liquid medium. (**G**) Transfer of cultured cells to a 3 L bioreactor. (**H**) One week after culture in the bioreactor. (**I**) Industrial bioreactors used for the large-scale production of AVPE. (**J**) Drying cells using a freeze-dryer. (**K**) Final product of the *Aloe vera* phytoplacenta freeze-dried cells, highlighting its texture and appearance.

For the production of Aloe vera phytoplacenta cell extracts in vitro, we prepared Aloe vera phytoplacenta cells as shown in Figure 1. Briefly, we obtained Aloe vera phytoplacenta from the Aloe vera flower (Figure 1A), which was further dissected into several parts (Figure 1B). We isolated the phytoplacenta of Aloe vera (Figure 1C) and cultured it in vitro for 1 to 2 months (Figure 1D). This was followed by suspension cell culture of the screened cell lines, which took 2 to 5 months (Figure 1F). Large-scale production of Aloe vera phytoplacenta cells was conducted using bioreactors for 4 to 6 weeks (Figure 1I). Finally, the cells were harvested and subjected to freeze-drying (Figure 1J). The harvested cells were further extracted by heating, and they could then be used for various applications in the pharmaceutical and cosmetic industries.

### 3.2. Cell Viability Assessment of HaCaT Cells Treated with AVPE Using CCK-8 Assay

**Figure 2:**
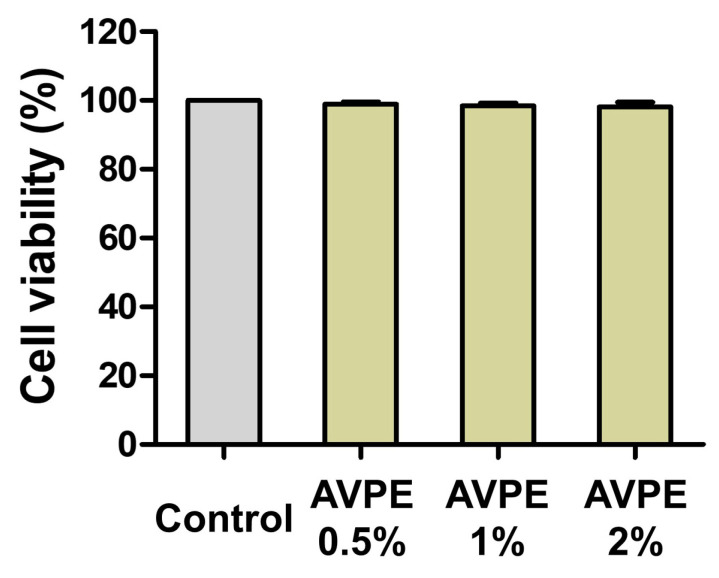
Cell viability of HaCaT cells treated with AVPE assessed by CCK-8 assay: control group with no treatment; 0.5%: HaCaT cells treated with 0.5% AVPE; 1%: HaCaT cells treated with 1% AVPE; 2%: HaCaT cells treated with 2% AVPE. The graph shows the percentage of viable cells for each treatment condition relative to the control.

We assessed the cell viability of HaCaT cells treated with AVPE at concentrations of 0.5%, 1%, and 2% using the CCK-8 assay (Figure 2) to evaluate toxicity, determine safe concentration ranges, and ensure extract quality and consistency. The results showed no cytotoxic effects at these concentrations: Control: 100%, 0.5% AVPE: 98.95%, 1% AVPE: 98.46%, and 2% AVPE: 98.15%. There were no significant differences between the three AVPE concentrations compared to the control group, indicating that AVPE is non-toxic to epidermal cells at the tested concentrations.

### 3.3. Effects of AVPE on Skin Moisturizing, Wound Healing, and Anti-Inflammatory Responses

**Figure 3:**
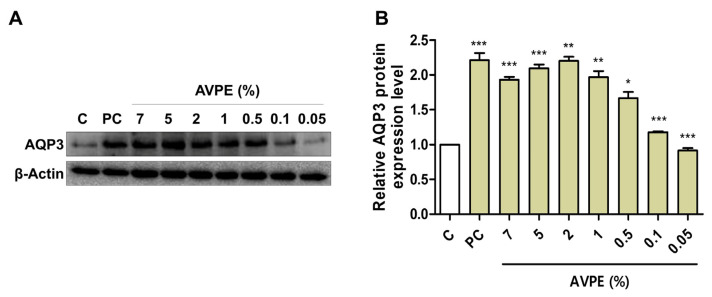
AVPE enhances skin moisturizing through increased AQP3 expression. Results of (**A**) Western blot analysis of AQP3 and β-actin protein levels in skin treated with different concentrations of AVPE (0.05%, 0.1%, 0.5%, 1%, 2%, 5%, and 7%) compared to control (C) using distilled water. Glyceryl glucoside (1%) was used as a positive control. β-actin served as a loading control. (**B**) Bar graph depicting the relative AQP3 protein expression levels in skin treated with AVPE. The expression level increases significantly in a dose-dependent manner, with a maximum 120% increase observed at the 2% AVPE concentration. Error bars represent standard deviation. *, **, and *** indicate *p* < 0.05, *p* < 0.01, and *p* < 0.001, respectively.

We examined AQP3 protein expression upon treatment with seven different concentrations of AVPE (7%, 5%, 2%, 1%, 0.5%, 0.1%, and 0.05%) by Western blot analysis (Figure 3). AQP3 serves as an important marker for skin moisturizing due to its crucial role in skin hydration and barrier function. The application of AVPE significantly increased the expression of AQP3 protein in the skin, indicating enhanced skin moisturizing properties compared to the control (Figure 3A). For instance, treatment with 2% AVPE resulted in a 120% increase in AQP3 protein expression compared to the control, with strong statistical support (*p* < 0.01) (Figure 3B). However, expression of AQP3 was slightly reduced after treatment with 5% and 7% AVPE compared to the 2% concentration.

**Figure 4:**
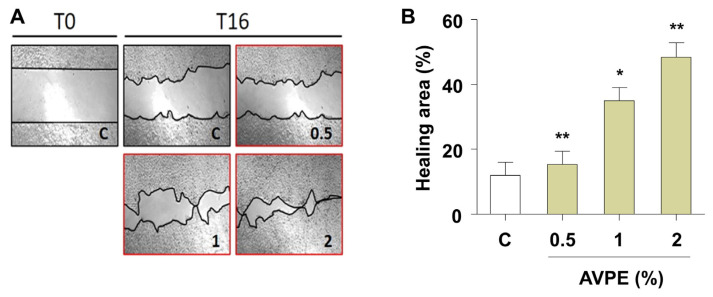
AVPE enhances wound healing. (**A**) Images showing the wound-healing process at time points T0 and T16 for skin treated with different concentrations of AVPE (0.5%, 1%, and 2%) compared to control (C). (**B**) Bar graph illustrating the percentage of the healing area in skin treated with AVPE. The healing area significantly increases in a dose-dependent manner, with a fourfold increase observed at the 2% AVPE concentration. Error bars represent standard deviation. * and ** indicate *p* < 0.05 and *p* < 0.01, respectively.

Next, we examined AVPE’s potential role in wound healing. AVPE treatment significantly enhanced wound healing at all tested concentrations (0.5%, 1%, and 2%). The healing area increased in a dose-dependent manner with increasing AVPE concentration (Figure 4A). Notably, treatment with 2% AVPE resulted in a fourfold increase in the healing area compared to the control, with strong statistical significance (*p* < 0.01) (Figure 4B). These results demonstrate that AVPE exhibits potent wound-healing properties.

**Figure 5:**
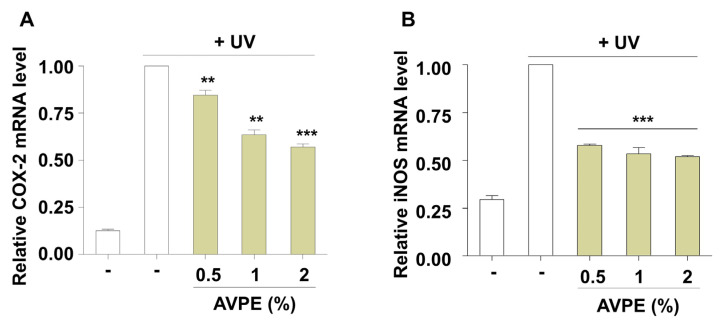
AVPE exhibits anti-inflammatory effects in UV-exposed skin. (**A**) Relative *COX-2* mRNA expression levels in UV-exposed skin treated with different concentrations of AVPE (0.5%, 1%, and 2%) compared to untreated control (-). (**B**) Relative *iNOS* mRNA expression levels in UV-exposed skin treated with different concentrations of AVPE (0.5%, 1%, and 2%) compared to untreated control (-). Data are presented as mean ± SD. ** *p* < 0.01, *** *p* < 0.001 vs. untreated control.

*COX-2* and *iNOS* are critical enzymes that play key roles in inflammatory processes. *COX-2* catalyzes prostaglandin production, while *iNOS* synthesizes nitric oxide, both significantly contributing to inflammatory responses. Our study demonstrates that AVPE treatment potently reduces the levels of these inflammatory markers in UV-exposed skin (Figure 5). Specifically, AVPE treatment led to a marked decrease in *COX-2* and *iNOS* messenger RNA (mRNA) levels, indicating its strong anti-inflammatory properties (Figure 5). At the highest concentration tested (2%), AVPE resulted in a 43% and 48% reduction in *COX-2* (Figure 5A) and *iNOS* (Figure 5B) mRNA levels, respectively, compared to the untreated control. This dose-dependent reduction in inflammatory mediators suggests that AVPE effectively modulates inflammatory pathways. These findings indicate that AVPE may offer protective mechanisms against UV-induced skin inflammation, potentially contributing to improved skin health and resilience against UV-induced cellular damage.

### 3.4. Comparison of Facial Skin Temperature Changes Induced by AVPE-Containing Test Product and Control Product

**Figure 6:**
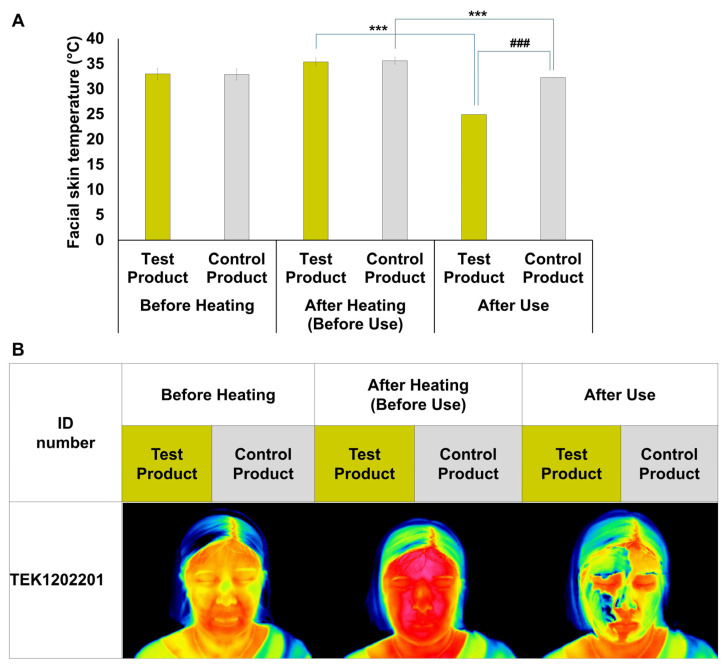
Effects of AVPE-containing test product and control product on facial skin temperature. (**A**) Bar graph showing the facial skin temperature (°C) of subjects before heating, after heating (before use), and after use of the test product (yellow bars) and control product (gray bars), with significant differences indicated by asterisks (*** *p* < 0.001) and hash marks (^###^ *p* < 0.001). (**B**) Thermal images of a representative subject (ID number TEK1202201) showing facial skin temperature before heating, after heating (before use), and after use of the test product (yellow labels) and control product (gray labels). The thermal images visually demonstrate the changes in facial skin temperature corresponding to the conditions shown in panel (**A**). Before heating: test product 34 °C, control product 34 °C; after heating: test product 37.20 °C, control product 37.20 °C; after use: test product 25.20 °C, control product 33.20 °C.

In the clinical trial evaluating the skin-soothing properties of the AVPE-containing test product, facial skin temperature was measured at three critical time points: before heating, after heat stimulation, and after product application (Figure 6). Before heating, both products showed similar baseline temperatures (test: 32.99 °C, control: 32.86 °C). After heat stimulation, temperatures increased to 35.4 °C and 35.64 °C for the test and control products, respectively. The most significant difference emerged after product application, with the AVPE-containing test product reducing facial skin temperature to 24.9 °C, compared to 32.3 °C for the control product (Figure 6A).

Thermal imaging of a representative subject (ID: TEK1202201) visually confirmed these temperature changes (Figure 6B). The images clearly depict facial skin temperature at all three stages, with the test product (yellow labels) showing a marked cooling effect after application and the control product (gray labels) exhibiting a less pronounced cooling effect.

### 3.5. Transcriptional Changes in HaCaT Cells Following AVPE Treatment Revealed by RNA-Seq Analysis

We investigated the molecular response of HaCaT cells to 1% AVPE using RNA-seq. Three biological replicates were analyzed, with sequence reads ranging from 13,352,211 to 18,043,731, demonstrating robust mapping to human reference transcripts at over 90% efficiency. Differential gene expression analysis was performed using DESeq2, employing specialized parameters to capture subtle transcriptional changes.

**Figure 7:**
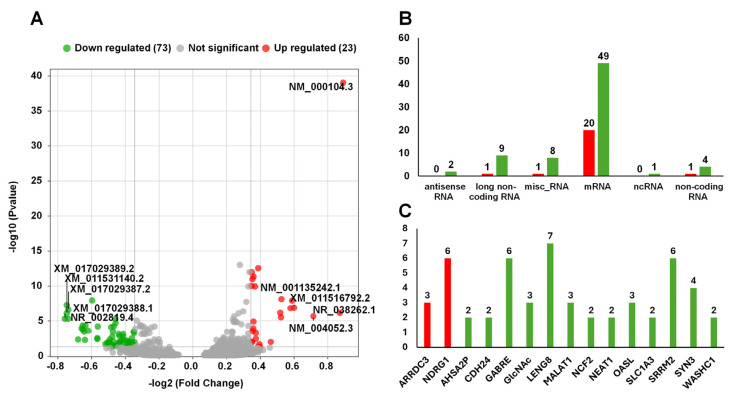
Results of the analysis of differential gene expression and RNA categorization: (**A**) Volcano plot of differential gene expression. Green dots represent downregulated RNAs (73 RNAs), gray dots represent non-significant RNAs, and red dots represent upregulated RNAs (23 RNAs). The x-axis represents the −log2 (fold change (FC)), and the y-axis represents the −log10 (*p*-value). Notable RNAs are labeled. (**B**) Number of RNAs according to categories. The categories include antisense RNA, long noncoding RNA, miscellaneous RNA, mRNA, ncRNA, and noncoding RNA. (**C**) Number of DERs derived from the same gene. Green and red colored bars indicate down- and upregulated RNAs, respectively.

The effects of AVPE on gene expression were subtle, resulting in a limited number of DERs when applying standard thresholds such as twofold changes and an adjusted *p*-value below 0.01. By relaxing the traditional stringent filtering criteria, we applied a more sensitive method to detect subtle transcriptional shifts that might have been overlooked using standard thresholds. We identified DERs using an FC threshold of greater than 1.3 for upregulation or less than 0.77 for downregulation, coupled with an adjusted *p*-value cutoff of 0.05. This refined analysis revealed 14 upregulated and 58 downregulated RNAs (Table S1, Figure 7A).

Our transcriptomic analysis of AVPE-treated HaCaT cells revealed subtle but significant changes in gene expression. We identified differentially expressed genes (DEGs) involved in various biological processes, including response to hydrogen peroxide, cell–cell adhesion, and synaptic transmission. Key findings include the modulation of genes related to epidermal differentiation, inflammation, and oxidative stress. Notably, AVPE upregulated some genes important for skin barrier function (e.g., *FLG*, *KLK14*, *CAPN1*) while downregulating others (e.g., *IVL*, *TGM2*, *CYP2E1*). Pathway analysis highlighted the involvement of AVPE in metabolic processes such as the pentose phosphate pathway and in signaling pathways like the GABAergic synapse pathway. These transcriptional changes provide insight into the molecular mechanisms underlying AVPE’s observed effects on skin hydration, anti-inflammatory activity, and overall skin health.

We further categorized the identified DERs according to their types (Figure 7B). The most abundant category was mRNAs, which included 20 upregulated and 49 downregulated transcripts. Additionally, we identified other RNA types with varying expression patterns, such as antisense RNA (two upregulated), long noncoding RNA (nine downregulated, one upregulated), miscellaneous RNA (eight downregulated, one upregulated), ncRNA (one upregulated), and noncoding RNA (one downregulated, four upregulated).

Interestingly, our analysis revealed multiple RNA transcripts originating from single genes (Figure 7C). We identified 15 genes that produced at least two DERs: *ARRDC3*, *NDRG1*, *AHSA2P*, *CDH24*, *GABRE*, *GlcNAc*, *LENG8*, *MALAT1*, *NCF2*, *NEAT1*, *OASL*, *SLC1A3*, *SRRM2*, *SYN3*, and *WASHC1*. Notably, *NDRG1*, *GABRE*, *LENG8*, and *SRRM2* exhibited exceptional diversity, each generating more than six distinct RNA transcripts.

### 3.6. Gene Ontology (GO) Enrichment Analysis of DERs

**Figure 8:**
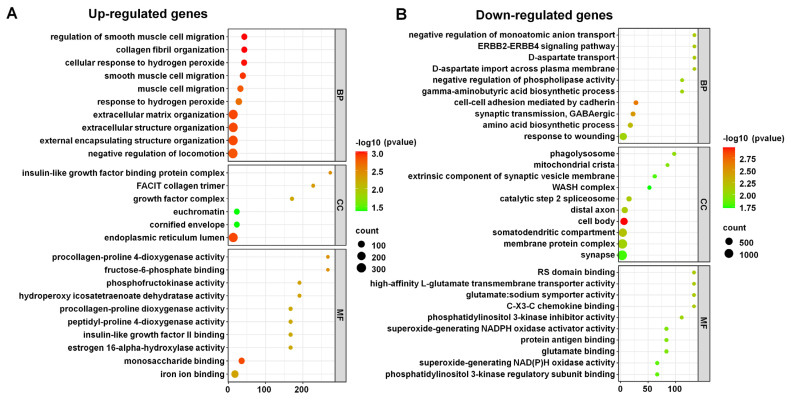
Gene ontology (GO) enrichment analysis of DERs. The identified enriched GO terms for upregulated RNAs (**A**) and downregulated RNAs (**B**). The dot plot shows the significantly enriched GO terms in the categories of biological process (BP), cellular component (CC), and molecular function (MF) for upregulated genes. The color of the dots represents the −log10 (*p*-value), and the size of the dots represents the number of genes associated with each GO term.

We conducted a GO enrichment analysis to investigate the biological functions, cellular components, and molecular activities associated with the DERs. We then categorized the results into two main groups, upregulated RNAs (Figure 8A) and downregulated RNAs (Figure 8B), each of which was further classified into three GO domains: biological process (BP), cellular component (CC), and molecular function (MF).

For upregulated RNAs, key processes involved response to hydrogen peroxide, muscle cell migration, and extracellular structure organization. Notable cellular components included the cornified envelope, euchromatin, and endoplasmic reticulum lumen. Important molecular functions highlighted were estrogen 16-alpha-hydroxylase activity, insulin-like growth factor II binding, and iron ion binding (Figure 8A).

The GO enrichment analysis for downregulated RNAs highlighted significant biological processes such as cell–cell adhesion mediated by cadherin, synaptic transmission (GABAergic), and amino acid biosynthesis (Figure 8B). Key signaling pathways included ERBB2–ERBB4. Notable cellular components involved the cell body, somatodendritic compartment, and membrane protein complex. Important molecular functions included chemokine binding, high-affinity L-glutamate transmembrane transporter activity, and superoxide-generating NADPH oxidase activator activity. These findings suggest that the upregulated RNAs play crucial roles in cell communication, signaling, and metabolic processes.

### 3.7. Pathway Enrichment Analysis of DERs in KEGG and Reactome Pathways

**Figure 9:**
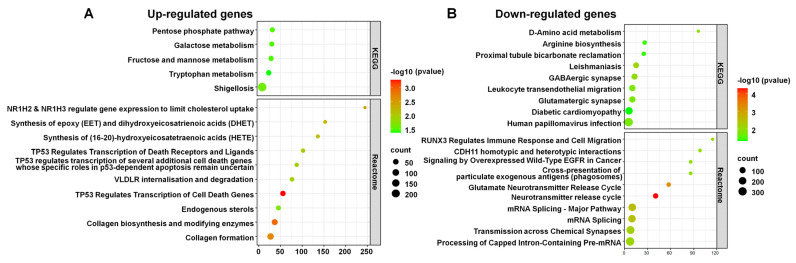
DERs in KEGG and Reactome pathways. The enriched pathways associated with upregulated RNAs (**A**) and downregulated RNAs (**B**). The x-axis represents the number of RNAs, while the y-axis represents the pathways. The color gradient from green to red indicates the −log10 (*p*-value), with red representing higher significance. The size of the dots corresponds to the RNA count within each pathway.

We analyzed DERs within various KEGG and Reactome pathways, highlighting those enriched with upregulated and downregulated RNAs (Figure 9). For upregulated RNAs in KEGG pathways, notable examples included the pentose phosphate, galactose metabolism, and tryptophan metabolism pathways (Figure 9A). In Reactome pathways, significant examples were TP53 regulating transcription of cell death genes, collagen biosynthesis, and modifying enzymes, and NR1H2 and NR1H3 regulating gene expression to limit cholesterol uptake.

Conversely, for downregulated RNAs, notable KEGG pathways included Leishmaniasis, GABAergic synapse, and D-Amino acid metabolism (Figure 9B). Significant Reactome pathways encompassed the neurotransmitter release cycle, the glutamate neurotransmitter release cycle, and mRNA splicing.

### 3.8. Effect of AVPE on Gene Expression Involved in Epidermal Differentiation, Inflammation, and Oxidative Stress Response

RNA-seq has the advantage of yielding global gene expression data from a single analysis. Based on the RNA-seq expression data, we examined changes in several important genes involved in epidermal differentiation, inflammation, and oxidative stress response.

**Figure 10:**
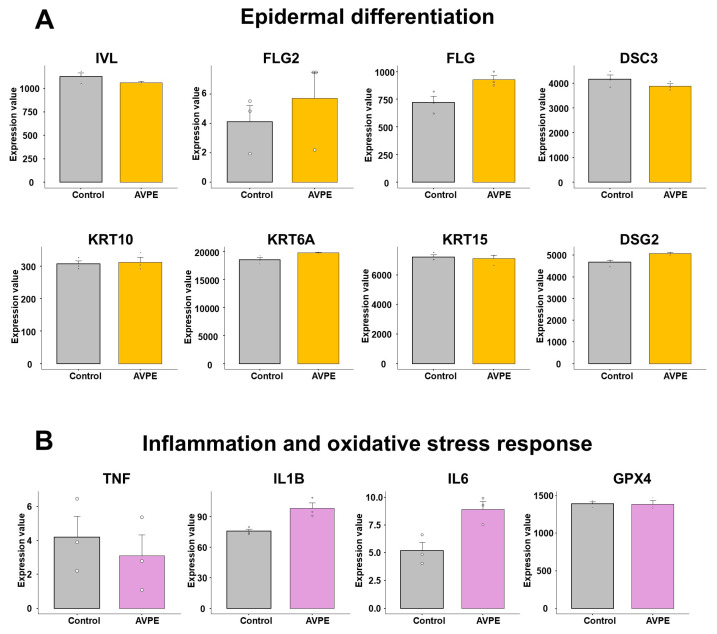
Effect of AVPE on gene expression related to epidermal differentiation, inflammation, and oxidative stress response. (**A**) Epidermal differentiation genes: *IVL*, *FLG2*, *FLG*, *DSC3*, *KRT10*, *KRT6A*, *KRT15*, and *DSG2*. (**B**) Inflammation genes: *TNF*, *IL1B*, and *IL6*, and oxidative stress gene: *GPX4*. The gene expression data were obtained from RNA-seq analysis, and normalized values were used for the comparison.

For epidermal differentiation, we selected eight genes: *IVL*, *FLG2*, *FLG*, *KRT10*, *KRT6A*, *KRT15*, *DSG2*, and *DSC3* (Table S2 and Figure 10A). Among these, the expression of *FLG2* was very low in HaCaT cells, followed by *KRT10*, based on the normalized expression value. In contrast, the expression of *KRT6A* was very high in HaCaT cells. In most cases, there were no significant differences between control and AVPE-treated samples. However, the expression levels of *IVL* and *DSC3* were slightly lower in AVPE-treated samples compared to the control, while those of *FLG2*, *FLG*, *KRT6A*, and *DSG2* were slightly higher in AVPE-treated samples compared to the control.

For genes related to inflammation, we initially selected 10 genes: *IL1B*, *IL6*, *TNF*, *IFNG*, *IL4*, *IL17A*, *IL22*, *IL25*, *TSLP*, and *IL33* (Table S2 and Figure 10B). Of these, only *TNF*, *IL1B*, and *IL6* were expressed in HaCaT cells, while the other genes were not expressed at all. Moreover, the expression levels of *TNF* and *IL6* were very low, with values less than 10 based on the normalized expression value. The expression of *TNF* decreased in AVPE-treated samples, while *IL1B* and *IL6* were upregulated in AVPE-treated samples. For oxidative stress response, we selected two genes: *GPX4* and *GPX5*. However, *GPX5* was not expressed at all, and there was no significant difference in the expression of *GPX4* between the two conditions.

### 3.9. Gene Expression Analysis of Cell Signaling Pathways and Other Functions in Control and AVPE-Treated Samples

**Figure 11:**
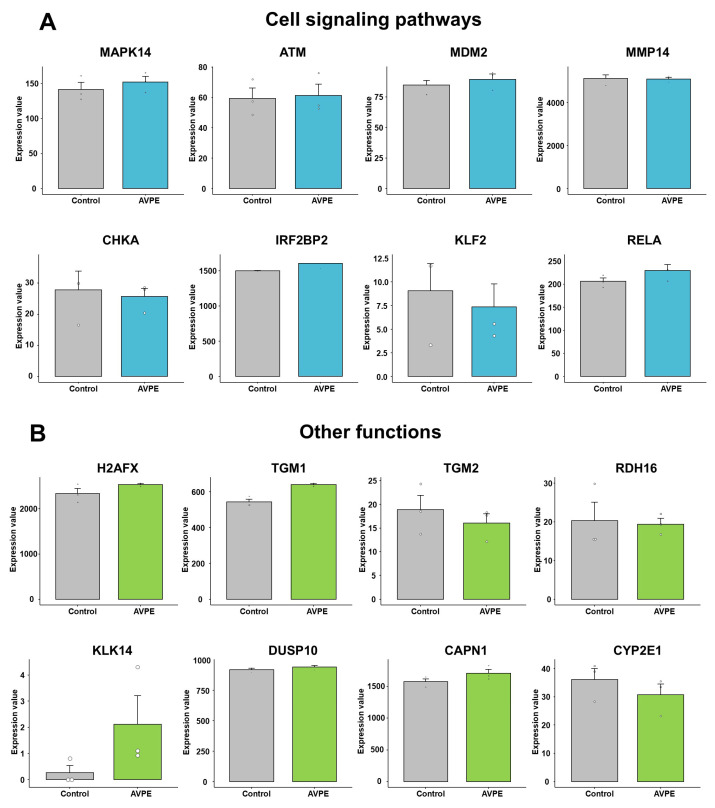
Effect of AVPE on expression of genes related to cell signaling pathways and other functions. (**A**) Genes related to cell signaling pathways: *MAPK14*, *ATM*, *MDM2*, *MMP14*, *CHKA*, *IRF2BP2*, *KLF2*, and *RELA*. (**B**) Genes related to other functions: *H2AFX*, *TGM1*, *TGM2*, *RDH16*, *KLK14*, *DUSP10*, *CAPN1*, and *CYP2E1*. The gene expression data were obtained from RNA-seq analysis, and normalized values were used for the comparison.

In our analysis of cell signaling pathways, we examined the expression of eight key genes: *MAPK14*, *ATM*, *MDM2*, *MMP14*, *CHKA*, *IRF2BP2*, *KLF2*, and *RELA* (Figure 11A). The expression levels significantly varied among these genes in HaCaT cells. For example, *KLF2* showed very low expression (normalized expression value < 10), while *MMP14* exhibited remarkably high expression (>4000) (Table S2 and Figure 11A). Although no statistically significant differences were observed between the control and AVPE-treated conditions, we noted some trends. That is, *MAPK14*, *MDM2*, *IRF2BP2*, and *RELA* showed upregulation in AVPE-treated samples, whereas *CHKA* and *KLF2* were downregulated.

Our investigation extended to genes involved in various skin-related functions (Table S2 and Figure 11B). Notably, *KLK14*, which plays a role in skin inflammation processes, showed low expression in untreated HaCaT cells but was significantly upregulated in AVPE-treated samples. We also examined genes crucial for tissue maintenance and cell differentiation, including *TGM1*, *TGM2*, and *DUSP10*, and found that *TGM1* was upregulated in AVPE-treated samples while *TGM2* was downregulated and *DUSP10* expression remained relatively constant across both conditions.

*H2AFX*, important for DNA double-strand break repair and protection against UV radiation damage, showed upregulation in AVPE-treated samples (Table S2 and Figure 11B). Similarly, *CAPN1*, which regulates cell adhesion and migration by affecting adhesion molecules and integrin-mediated functions, was also upregulated in such samples.

*RDH16*, a key enzyme in the biosynthesis of retinoic acid essential for skin health, did not show significant changes in expression with AVPE treatment (Table S2 and Figure 11B). Interestingly, *CYP2E1*, involved in generating reactive oxygen species (ROS), was downregulated in AVPE-treated samples. This downregulation might decrease oxidative stress in skin cells, potentially offering protection against cellular damage.

## 4. Discussion

Traditionally, *Aloe vera* propagation has relied on slow and costly vegetative methods using suckers or lateral shoots [35]. While previous research has explored tissue culture for rapid *Aloe vera* production [36,37], to our knowledge, no studies have demonstrated the large-scale generation of *Aloe vera* cells in vitro. Furthermore, we focused on phytoplacenta, a component present in minute quantities within the whole plant, making it challenging to obtain. To address the scarcity of phytoplacenta materials, we adapted plant cell culture technology for mass production. Our groundbreaking study showcases the potential of plant cell culture in producing AVPE, marking the first instance of large-scale in vitro AVPE production.

Given that phytoplacenta originates from flower tissue, *Aloe vera* phytoplacenta and flower tissue may share similar components. Despite having established a reproducible AVPE production method here, we acknowledge the need for comprehensive chemical characterization. Our future research will focus on detailed compositional analysis, using techniques such as HPLC-MS, to fully identify AVPE components and ensure batch-to-batch consistency, thus strengthening the reproducibility and reliability of our findings.

Our study on AVPE reveals promising results across multiple aspects of skin health, supported by previous scientific literature. The cell viability results align with earlier studies on *Aloe vera* extracts, which demonstrated antibacterial activity against various pathogens without causing toxicity [38]. This is in line with our finding that AVPE is non-toxic to epidermal cells at concentrations up to 2%.

Regarding skin moisturizing effects, our results showing increased AQP3 expression are consistent with prior research on *Aloe vera*’s hydrating properties. The previous study found high levels of hyaluronic acid and dermatan sulfate, compounds known to enhance skin hydration and collagen synthesis, in granulation tissue treated with *Aloe vera*, which contributed to improved wound healing [39]. 

The wound-healing properties of AVPE observed in our study are supported by multiple prior investigations. Oryan et al. reported that topical *Aloe vera* application on mice skin wounds modulated inflammation, stimulated fibroplasia, and increased collagen production [39]. Similarly, Khorasani et al. found that *Aloe vera* cream accelerated burn wound healing compared to silver sulfadiazine, with 100% of *Aloe vera*-treated burns healing within 19 d [40].

Our findings on AVPE’s anti-inflammatory effects are also corroborated by earlier research. Panahi et al. demonstrated that *Aloe vera* gel combined with olive oil was more effective than phenytoin cream in reducing pain and promoting wound healing in patients with chronic wounds [41]. This aligns with our observations of reduced *COX-2* and *iNOS* expression in UV-exposed skin treated with AVPE.

The skin-soothing properties of the AVPE-containing product observed in our clinical trial are consistent with *Aloe vera*’s known cooling effects. Avijegan et al. reported significantly faster wound healing and reduced hospitalization time in patients treated with *Aloe vera* gel compared to conventional treatments [42], which aligns with our findings on AVPE’s cooling effect on facial skin temperature. These collective findings from our study and previous research underscore the multifaceted benefits of AVPE for skin health, including its moisturizing, wound-healing, anti-inflammatory, and skin-soothing effects. Given the non-toxic nature of AVPE, combined with its diverse beneficial properties, it is a promising ingredient for skincare formulations targeting various aspects of skin health and protection. While our study provides promising insights into AVPE’s potential, we acknowledge its limitations, particularly the small sample size. Larger clinical trials are necessary to validate these findings and fully explore AVPE’s efficacy in diverse populations over extended periods.

Our RNA-seq analysis of 1% AVPE-treated HaCaT cells revealed subtle yet significant transcriptional changes, providing insights into the molecular mechanisms underlying AVPE’s effects on skin health. This approach aligns with previous studies that have used RNA-seq to investigate the effects of natural compounds on skin cells. For instance, transcriptome analysis using RNA-seq demonstrated that *Hibiscus sabdariffa* plant extract (HSPE) induced more substantial changes in human skin cells compared to *Hibiscus sabdariffa* callus extract (HSCE), with HSPE upregulating genes related to angiogenesis, oxidation reduction, and glycolysis, and HSCE primarily increasing the expression of ribosomal proteins and IFI6, which is associated with healing radiation-injured skin cells [25].

The GO enrichment analysis of DERs highlighted several key biological processes affected by AVPE treatment, including responses to hydrogen peroxide and extracellular structure organization. These findings are consistent with previous research on *Aloe vera*’s effects on skin. For example, Surjushe et al. reported that *Aloe vera* contains antioxidants such as glutathione peroxidase and superoxide dismutase, which can help protect against oxidative stress [1]. The upregulation of genes involved in extracellular matrix organization corroborates prior findings that *Aloe vera* treatment enhances collagen synthesis and crosslinking during wound healing [43].

Our pathway enrichment analysis revealed that AVPE influences various cellular processes, including the pentose phosphate and galactose metabolism pathways. These findings are supported by previous studies on *Aloe vera*’s metabolic effects. One study demonstrated that *Aloe vera* gel contains various monosaccharides and polysaccharides that can influence cellular metabolism [44]. The downregulation of inflammatory markers such as *TNF* in our study is consistent with the anti-inflammatory properties of *Aloe vera* reported in earlier research [2]. Of these, only *TNF*, *IL1B*, and *IL6* were expressed in HaCaT cells, while the other genes were not expressed at all.

The subtle changes observed in genes related to epidermal differentiation, such as the upregulation of *FLG* and *KRT6A*, align with previous research on *Aloe vera*’s effects on skin barrier function. For example, one study showed that *Aloe vera* extract improved skin hydration, possibly by enhancing the expression of skin barrier proteins [45]. These molecular insights help to elucidate the mechanisms underlying *Aloe vera*’s well-documented benefits in skincare, including improved wound healing and reduced inflammation. While this RNA-seq analysis revealed potential molecular mechanisms of AVPE in HaCaT cells, including changes in genes involved in skin barrier function and keratinization, these observations were based on mRNA levels. Protein level validation is required to confirm these findings. Due to resource limitations, Western blot analysis for DEGs, including *FLG* and *KRT6A*, was not performed in this study. In the future, we will address this limitation by investigating corresponding protein expression levels to provide a more comprehensive understanding of AVPE’s mechanism of action.

## 5. Conclusions

Our study demonstrated AVPE’s therapeutic potential for skin health through in vitro, in vivo, and transcriptomic analyses. AVPE, produced via a robust in vitro system, exhibited no cytotoxicity in HaCaT cells. It significantly enhanced skin hydration by increasing AQP3 expression and demonstrated potent anti-inflammatory activity by reducing *COX-2* and *iNOS* levels in UV-exposed skin. Clinical trials confirmed its skin-soothing properties, showing a significant reduction in skin temperature after heat stimulation. RNA-seq revealed subtle but significant gene expression changes, with DERs involved in various biological processes, including response to hydrogen peroxide, cell–cell adhesion, and synaptic transmission. Pathway analysis highlighted involvement in pathways such as the pentose phosphate and GABAergic synapse pathways. AVPE modulated genes related to epidermal differentiation, inflammation, and oxidative stress, upregulating some (e.g., *FLG*, *KLK14*, *CAPN1*) while downregulating others (e.g., *IVL*, *TGM2*, *CYP2E1*). These findings suggest AVPE’s multifaceted benefits for skin health, including hydration, anti-inflammatory effects, skin soothing, and modulation of key genes and pathways. Given AVPE’s demonstrated benefits in skin hydration, anti-inflammatory effects, and gene modulation, our findings suggest its potential as a valuable ingredient in commercial skincare products. However, further research and development are needed to optimize formulation, assess long-term efficacy, and ensure safety for widespread use. Taken together, they indicate that AVPE shows promise as a valuable ingredient for skincare products.

## Data Availability

The sequencing data generated in this study have been deposited in the NCBI SRA database (accession numbers: SRR32174142–SRR32174147).

## Supplementary Materials

The following supporting information can be downloaded at: https://www.mdpi.com/article/10.3390/life15030397/s1, Table S1: Differentially expressed RNAs and their functional categories; Table S2: Differential gene expression analysis results for selected genes in human keratinocytes treated with AVPE.

## Abbreviations

The following abbreviations are used in this manuscript:

AVPE	Aloe vera phytoplacenta extract
KEGG	Kyoto Encyclopedia of Genes and Genomes
GO	Gene Ontology
